# Metabolic Disturbances of a High-Fat Diet Are Dependent on APOE Genotype and Sex

**DOI:** 10.1523/ENEURO.0267-19.2019

**Published:** 2019-10-14

**Authors:** Nahdia S. Jones, Katarina Q. Watson, G. William Rebeck

**Affiliations:** Department of Neuroscience, Georgetown University, Washington, DC 20007

**Keywords:** apolipoprotein E, diet, metabolism, mouse model, obesity

## Abstract

Apolipoprotein E4 (*APOE4*) is the strongest genetic risk factor for Alzheimer’s disease (AD). *APOE4* is also associated with an increased risk of metabolic syndrome. Obesity is a major environmental risk factor for AD. While *APOE* genotype and obesity independently affect metabolism and cognition, they may also have synergistic effects. Here, we examined the metabolic and behavioral alterations associated with a high-fat diet (HFD) in male and female *APOE* knock-in mice. Male and female mice were fed a 45% kcal HFD or a 10% kcal low-fat diet (LFD) for 12 weeks and adipose tissue accumulation, glucose levels, anxiety-like behavior, and spatial memory were examined. We found that with HFD, male *APOE4* mice were more susceptible to metabolic disturbances, including visceral adipose tissue (VAT) accumulation and glucose intolerance when compared to *APOE3* mice, while female *APOE3* and *APOE4* mice had similar metabolic responses. Behaviorally, there were no effects of HFD in mice of either genotype. Our results suggest that metabolic responses to HFD are dependent on both sex and APOE genotype.

## Significance Statement

Apolipoprotein E4 (*APOE4*) and obesity are independently associated with increased risk of metabolic syndrome and cognitive impairment. Obesity may cause greater metabolic and cognitive disturbances in *APOE4* carriers. However, the metabolic and cognitive effects of obesity on male and female *APOE4* carriers remain unknown. Here, we examine and compare the metabolic and cognitive disturbances caused by a high-fat diet (HFD) in both male and female *APOE3* and *APOE4* mice. Through this study, we examine how HFD affects the *APOE3* and *APOE4* genotype and how these effects differ across sexes.

## Introduction

Apolipoprotein E4 (*APOE4*) is the strongest genetic risk factor for Alzheimer’s disease (AD; Huang et al., 2004; Raber et al., 2004). In the periphery, APOE is a component of lipoproteins responsible for the metabolism of plasma lipids. Through binding to different lipoprotein receptors, APOE traffics high-density lipoproteins (HDLs) and very low-density lipoproteins (VLDLs) throughout the body for storage or elimination (Huang and Mahley, 2014). In the CNS, APOE-HDL are responsible for trafficking lipids from astrocytes to neurons and for clearance into the circulation (Liu et al., 2013). There are three *APOE* alleles, *APOE2*, *APOE3*, and *APOE4*, and each allele is associated with a differential risk of AD. *APOE2* has an allele frequency of 8% in the United States and is associated with a 40% decreased risk of developing AD (Huang and Mahley, 2014). *APOE3* has an allele frequency of 77%; homozygous *APOE3* carriers (64% of the population) are defined as having a normal risk of AD (Liu et al., 2013). *APOE4* has an allele frequency of 15%; heterozygous carriers are 2.3 times more likely to develop AD and homozygous carriers are 14 times more likely (Liu et al., 2013).

Obesity and metabolic syndrome are also major risk factors for AD. Obesity is a medical condition characterized by increased body mass index (BMI) and currently affects 40% of adults and 20% of children in the United States (Hales et al., 2017). In the periphery, obesity can lead to metabolic syndrome including increases in visceral adipose tissue (VAT) and subcutaneous adipose tissue (SAT), and decreases in glucose metabolism and insulin sensitivity (Mathieu et al., 2009; Neth and Craft, 2017). In the CNS, obesity is associated with increased inflammation, deficits in cognitive functioning, mild cognitive impairment, and AD (Gustafson et al., 2009; Besser et al., 2014; Bloor and Symonds, 2014).

While *APOE* genotype and obesity independently affect AD risk, they may also have combined effects. *APOE4* is associated with increased cognitive deficits and increased risk of metabolic syndrome (Arbones-Mainar et al., 2008; Rodriguez et al., 2013; Torres-Perez et al., 2016), which are exacerbated when combined with obesity. Obese *APOE4* carriers can have elevated glucose and insulin levels (Elosua et al., 2003), and deficits in cognitive functioning (Ghebranious et al., 2011; Zade et al., 2013). Data in humans is complemented by mouse models. *APOE4* knock-in mice have increased insulin resistance and deficits in glucose metabolism when on high-fat diets (HFDs; Arbones-Mainar et al., 2008; Johnson et al., 2017). Cognitive performance of *APOE4* mice on HFDs have shown mixed results, with either increased deficits in spatial memory (Johnson et al., 2017) or no cognitive differences (Janssen et al., 2016). Here we compare the effects of a HFD, with macronutrients equivalent to a western diet, on male and female homozygous *APOE3* and *APOE4* mice. We examined both metabolic and behavioral alterations and found that HFD increases metabolic disturbances in both *APOE3* and *APOE4* mice, with *APOE4* mice being more robustly affected. We also found that male and female mice differentially respond to HFD.

## Materials and Methods

### Animals/diet

Male and female human *APOE3* and *APOE4* knock-in mice on a C57BL/6J background (*n* = 5–9/sex; the gift of Patrick Sullivan) were fed either a HFD (45% kcal fat, Research Diets-D12451) or ingredient matched low-fat diet (LFD; 10% kcal fat, Research Diets-D12450H) for 12 weeks beginning at six months of age. Food and water were provided *ad libitum* and mice were weighed weekly during the 12 weeks. At the end of the 12 weeks mice underwent glucose tolerance testing (GTT), abdominal and neck MRI, and behavioral assays which occurred over a two-week period (12–14 weeks). The mice remained on the diets throughout the GTT, MRIs, and behavioral assays. All experiments followed the guidelines of the Institutional Animal Care and Use Committee.

### Glucose testing

Mice were restricted from food for 6 h before the measures of baseline glucose levels and glucose tolerance to a glucose bolus. Fasting baseline glucose levels were followed by an intraperitoneal injection of 20% glucose (1 mg/kg). Blood glucose levels from tail vein withdrawal were measured using the AccuChek Guide glucose meter at 15, 30, 60, and 120 min after injection.

### MRI

After completion of behavioral assays, mice underwent small animal imaging in the Preclinical Imaging Research Laboratory at the Georgetown University Medical Center. Mice were anesthetized using 3–5% isoflurane and maintained with 1–3% isoflurane. Images were taken with a 7-Tesla horizontal Bruker spectrometer run by Paravision 5.1; body temperature, heart rate, and respiration were monitored throughout the scan. Images were obtained for the abdominal white adipose tissues (WATs) VAT and SAT, and for the neck brown adipose tissue (BAT). Z-stack images were analyzed with ImageJ. VAT and SAT images were quantified as ratios of abdominal adipose tissue to abdominal organs (referred to as “body”). For BAT, images were quantified as the ratio of BAT intensity to the WAT intensity. The BAT and WAT intensities were measured using the mean gray value in ImageJ, with the darker areas being reflected as higher mean gray values indication higher BAT intensities. Higher BAT intensity indicates more BAT, which has the ability to convert excess food energy into thermal energy (Schulz and Tseng, 2013).

### Behavioral assays: open field test (OFT), elevated zero maze (EZM), and Barnes maze (BM)

For all behavioral assays, mice were placed in the behavioral suite for a 30-min acclimation period.

#### OFT

Mice freely explored a square (43 × 43 × 30 cm) open field apparatus for 300 s. During free exploration, locomotor activity and anxiety-like behaviors were recorded. The apparatus was divided into an inner zone and a bordering outer zone that lined the apparatus’s walls. Mice were placed in the center of the inner zone and behavior was recorded for the duration of the test. Behavior was recorded with Med Associates Activity Monitor 7. For locomotion, average speed (m/s) was assessed. For anxiety-like behavior, time spent in the inner versus outer zone (9 × 9 cm) was assessed as increased time in the outer zone indicating increased anxiety (Seibenhener and Wooten, 2015). Data were analyzed with GraphPad Prism 8.

#### EZM

Mice were exposed to a circular elevated zero apparatus (50 cm from floor, 50 cm in diameter, and 15-cm high closed regions) for 300 s of free exploration. The apparatus consists two closed regions and two open regions of equal sizes. Mice were placed on the center of an open region to begin testing and behavior was recorded for the duration of the 300 s using ANY-maze Behavioral tracking software 6.0. Time spent in the closed versus open regions of the apparatus was examined as a measure of anxiety and willingness to explore. Data were analyzed with GraphPad Prism 8.

#### BM

Mice were exposed to the BM for five consecutive days to test spatial learning and memory, as described (Speidell et al., 2019). The maze was present in a brightly lit room and 90-dB white noise. Mice were habituated to the maze on day 1, and then had four consecutive training days. During training days, mice underwent four trials with 15 min between each trial, and latency to first nose poke and latency to enter the escape hole (latency to escape) were recorded to examine spatial memory. Mice were recorded with ANY-maze Behavioral tracking software 6.0, and data were analyzed with GraphPad Prism 8.

### Statistics

All data are expressed as mean ± SD with the exception of behavioral assays which are expressed as mean ± SE. Comparisons between genotype, sex and diet were analyzed by three-way ANOVAs with Tukey’s multiple comparison test. Comparisons between genotype and sex were analyzed by two-way ANOVAs with Sidak’s multiple comparison test. Statistical significance was determined by a probability error of *p* < 0.05. All analyses were done using GraphPad Prism 8.

## Results

### HFD increases the weight of *APOE3* and *APOE4* mice

**Figure 1.**
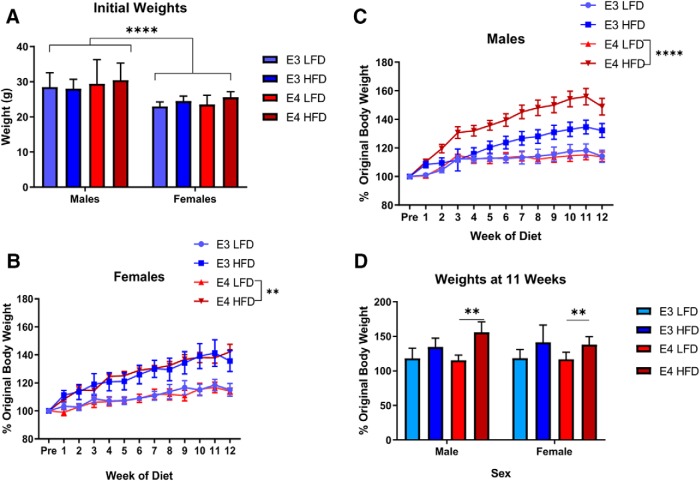
HFD increases weight gain and glucose intolerance. Weight gain comparison from pre-diet to week 12 on diet across *APOE* genotypes and sex. Initial weights of all mice in grams (***A***). Diet associated weight gain of female *APOE3* and *APOE4* mice (***B***) and male *APOE3* and *APOE4* mice (***C***). Direct comparison of male and female weight gain at week 11 on diet (***D***). E3 LFD: light blue, *APOE3* mice on a LFD, E3 HFD: dark blue, *APOE3* mice on a HFD, E4 LFD: light red, *APOE4* mice on a LFD, E4 HFD: dark red, *APOE4* mice on a HFD. Three-way ANOVA Tukey’s multiple comparison test, *N* = 5–9. ***A***, ****p < 0.0001; ***B***, **p < 0.003; ***C***, ****p < 0.0001; ***D***, **p < 0.0046.

To examine how the different *APOE* genotypes respond to obesity, we used a diet induced obesity model. Male and female mice (six months old) were placed on a HFD for 12 weeks and weighed weekly. At six months old, the male mice (across genotypes) weighed significantly more than female mice (*p* < 0.0001; Fig. 1*A*). To directly compare weight gain trajectories, weights were calculated as a percentage of each mouse’s original body weight. All groups gained weight over the course of the experiment, with HFD groups gaining more weight compared to LFD groups (Fig. 1).

In female mice, HFD resulted in a 40% increase from original body weight by week 11 in both *APOE3* and *APOE4* mice (*p* < 0.0001); both genotypes gained weight at the same rate. HFD mice also weighed more than LFD mice (*APOE3*: *p* < 0.06, *APOE4*: *p* < 0.005). Female *APOE3* and *APOE4* mice on the LFD experienced slight weight gain (15%, *p* > 0.8; Fig. 1*B*).

In male mice, HFD resulted in a 30% increase from original body weight in *APOE3* mice by week 11 (*p* < 0.0001) and resulted in a 45% increase from original body weight in *APOE4* mice by week 11 (*p* < 0.0001), although the differences between *APOE3* and *APOE4* genotypes were not statistically significant (*p* = 0.15). *APOE3* and *APOE4* mice on the LFD experienced slight weight gain (17%, *p* > 0.8; Fig. 1*C*). HFD *APOE3* mice did not weigh significantly more than the LFD *APOE3* mice (*p* = 0.57); HFD *APOE4* mice did weigh significantly more than LFD *APOE4* mice (*p* < 0.0001).

Across sexes, the weight gain due to HFD did not differ. However, while male *APOE3* did respond to HFD, they gained 15% less weight than male *APOE4* mice or female mice (Fig. 1*D*). On week 12, there was a slight decrease in body weight associated with the beginning of the metabolic and behavioral assays; therefore, statistical tests were conducted on data from week 11.

### HFD increases baseline glucose levels and glucose intolerance in *APOE3* and *APOE4* mice

**Figure 2.**
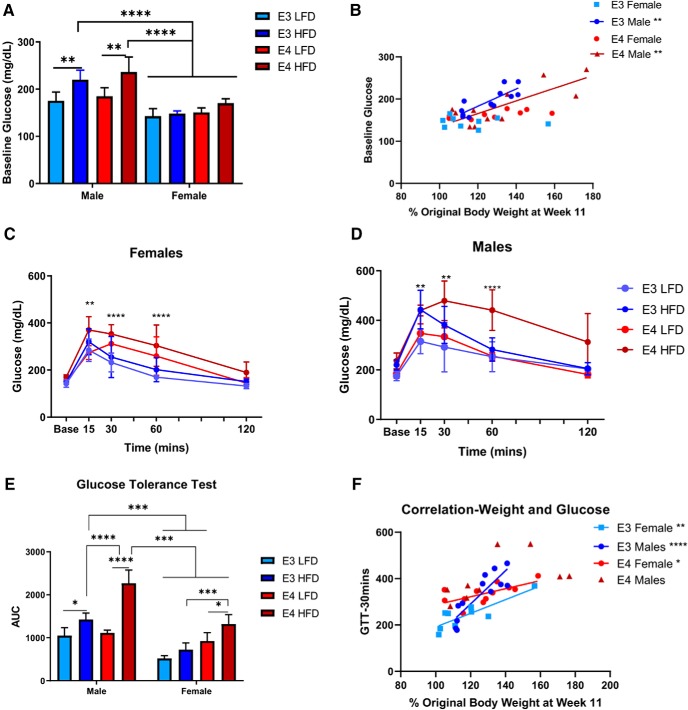
HFD increases baseline glucose and glucose intolerance. Comparison of baseline glucose levels in male and female *APOE3* and *APOE4* mice, first comparing within sex differences then across sex differences (***A***). Correlational analyses between weight, glucose levels, and sex. Lines indicate significant correlations (***B***). GTT in female (***C***) and male (***D***) *APOE3* and *APOE4* mice. Area under the curve (AUC), complete deviation from baseline glucose levels, comparing within sex differences and across sex differences (***E***). Correlation between genotype, weight gain, sex, and glucose levels at 30 min. Lines indicate significant correlations (***F***). ***A***, ***C–E***, *N* = 4–6. Three-way ANOVA Tukey’s multiple comparison test. ***A***, **p < 0.002; ****p < 0.0001; ***C***, **p < 0.003 all groups deviate from baseline at 15 min, ****p < 0.0001 HFD and LFD *APOE4* deviates from baseline at 30 and 60 min; ***D***, **p < 0.002 all groups deviate from baseline at 15 min, *p < 0.02 all groups deviate from baseline at 30 min, ***p < 0.0002 HFD *APOE4* deviates from baseline at 60 min; ***E***, *p < 0.02, **p < 0.01, ***p < 0.0003, ****p < 0.0001. ***B***, ***F***, Linear regression. ***B***, *APOE3* males (*N* = 12): *R* ^2^ = 0.68, *p* = 0.001. APOE4 males (*N* = 11): *R* ^2^ = 0.63, *p* = 0.004. ***F***, *APOE3* males (*N* = 12): *R* ^2^ = 0.75, *p* = 0.0002. *APOE3* females (*N* = 9): *R* ^2^ = 0.72, *p* = 0.004. *APOE4* females (*N* = 12): *R* ^2^ = 0.42, *p* = 0.02.

Deficits in glucose metabolism are also associated with HFD. These deficits can lead to Type II diabetes and cognitive deficits. To test whether our model alters glucose metabolism, baseline glucose levels and glucose tolerance were measured after 12 weeks of HFD (Fig. 2).

In females, HFD *APOE3* and *APOE4* mice had similar baseline glucose levels; these levels did not differ from LFD *APOE3* and *APOE4* mice. In males, HFD *APOE3* and *APOE4* mice had similar baseline glucose levels; however, their levels were elevated when compared to LFD *APOE3* and *APOE4* mice (*p* < 0.002; Fig. 2*A*). Across sexes, male HFD mice had significantly higher baseline glucose levels than the female HFD mice (*p* < 0.0001; Fig. 2*A*). We reasoned that increased baseline glucose may be associated with weight gain, given the disparate levels of weight gains in male versus female mice. To test this hypothesis, we determined the correlation of weight gain with baseline glucose across genotype and sex. *APOE3* and *APOE4* weight gain was positively correlated with increased baseline glucose in males, but not females (*APOE3*: *p* = 0.001, *R* ^2^ = 0.68, *APOE4*: *p* = 0.003, *R* ^2^ = 0.62; Fig. 2*B*).

After baseline glucose levels, mice underwent GTT as a measurement of glucose metabolism. A bolus of glucose was given, and glucose levels measured at 15, 30, 60, and 120 min. In females, when compared to baseline, there was an increase in glucose levels in the first 15 min in all groups (*p* < 0.003). This increase remained in the HFD groups at 30 min (*p* < 0.0001), and 60 min (*p* < 0.0001; Fig. 2*C*). In males, when compared to baseline, there was an increase in glucose levels in the first 15 min and remained elevated at 30 min in all groups (*p* < 0.02). At 60 min, all mice returned to the range of baseline glucose except for HFD *APOE4* mice (*p* < 0.002; Fig. 2*D*). This indicates that the HFD *APOE4* mice did not metabolize the glucose as quickly or efficiently as the HFD *APOE3* mice or the LFD *APOE4* mice.

To examine overall differences in glucose tolerance over time across genotype and sex, we analyzed area under the curve in the GTT. In females, HFD *APOE4* mice had a larger deviation in glucose than HFD *APOE3* mice (*p* < 0.0003) and LFD *APOE4* mice had a larger deviation in glucose than LFD *APOE3* mice (*p* < 0.02). HFD *APOE4* mice also had a larger deviation than LFD *APOE4* mice (*p* < 0.02). This difference was not seen when comparing HFD *APOE3* mice and LFD *APOE3* mice (Fig. 2*E*). In males, HFD *APOE4* mice had a larger deviation in glucose than HFD *APOE3* mice (*p* < 0.0001). HFD mice also had a larger deviation in glucose than both LFD *APOE*3 mice (*p* < 0.05), and *APOE4* mice (*p* < 0.0001; Fig. 2*E*). Across sexes, glucose deviations in male HFD *APOE4* mice were greater than deviations seen in all female groups (*p* < 0.0003). Deviations in male HFD groups were larger than deviations seen in the female groups except female HFD *APOE4* mice (*p* < 0.003; Fig. 2*E*).

To test whether the glucose intolerances found could result from weight gains, we ran correlational analyses comparing weight gain with glucose levels 30 min after bolus. In both *APOE3* and *APOE4* mice, an increase in weight was positively correlated with higher glucose levels (*p* < 0.007), indicating any increase in weight may strongly affect glucose intolerance. There was a stronger positive correlation between weight gain and glucose intolerance in *APOE3* mice (*p* = 0.01) indicating weigh gain can drive glucose intolerance in *APOE3* mice while *APOE4* mice are more susceptible to glucose intolerance at lower weights (Fig. 2*F*). Glucose levels were significantly correlated with weight gain regardless of sex with the exception of *APOE4* males (*p* < 0.02; Fig. 2*F*).

### HFD increases VAT and SAT in *APOE3* and *APOE4* mice

**Figure 3.**
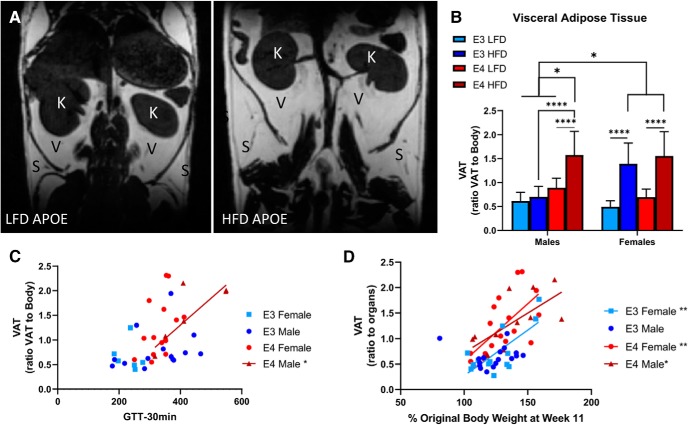
HFD increases VAT in *APOE3* and *APOE4* mice. Representative image of VAT and SAT in LFD mouse and HFD mouse. S, SAT; V, VAT; K, kidneys (***A***). Within sex and across sex quantification of VAT in *APOE3* and *APOE4* mice (***B***). Correlation of glucose intolerance and VAT accumulation across *APOE* genotypes and across sex. Lines indicate significant correlations (***C***). Correlation of weight gain and VAT accumulation across *APOE* genotypes and across sex. Lines indicate significant correlations (***D***). ***B***, *N* = 5–9. Within sex comparison: ****p < 0.0001, three-way ANOVA Tukey’s multiple comparison test. Across sex comparison: *p < 0.02, three-way ANOVA Tukey’s multiple comparison test. ***C***, ***D***, Linear regression analyses. ***C***, *APOE3* female (*N* = 6): *R* ^2^ = 0.52, p = 0.005; *APOE4* male (*N* = 7): *R* ^2^ = 0.48, *p* = 0.01; *APOE4* female (*N* = 14): *R* ^2^ = 0.43, *p* = 0.003. ***D***, *APOE4* male (*N* = 14): *R* ^2^ = 0.62, *p* = 0.03.

A metabolic disturbance associated with HFD is increased adipose tissue. SAT is the adipose tissue more associated with obesity; however, VAT is more noxious due to its direct contact with the organs and its ability to release inflammatory cytokines (Hotamisligil et al., 1995). To test whether our model results in increases in specific types of adipose tissue, we used small rodent MRIs to examine both VAT and SAT levels (Fig. 3*A*).

In females, HFD caused an increase in VAT compared to LFD (*p* < 0.0001; Fig. 3*B*). In males, HFD *APOE4* mice accumulated more VAT than LFD *APOE4* mice (*p* < 0.0001), but there was no similar effect for *APOE3* mice (*p* = 1.0; Fig. 3*B*). Across sexes, HFD mice had similar elevated VAT levels, except for the male *APOE3* mice, which did not differ from LFD mice (*p* < 0.02; Fig. 3*B*).

We analyzed the correlation between VAT and GTT, including the possible effects of genotype, sex, and diet. There was no correlation between VAT and GTT when considering genotype and diet (Fig. 3*C*); however, there was a correlation between VAT and GTT only in male *APOE4* mice (*R* ^2^ = 0.6, *p* = 0.03; Fig. 3*C*). We also ran correlational analyses comparing weight gain and VAT to see whether VAT was a large contributor to the weight gain. VAT and weight gain in *APOE3* females and *APOE4* mice positively correlated (*APOE3*: *R* ^2^ = 0.52 *p* = 0.005, *APOE4*: *R* ^2^ = 0.40, *p* = 0.01); however, there was not a positive correlation between VAT and weight gain in *APOE3* males (Fig. 3*D*). These findings indicate that VAT may act as a contributor to weight gain and glucose intolerance.

**Figure 4.**
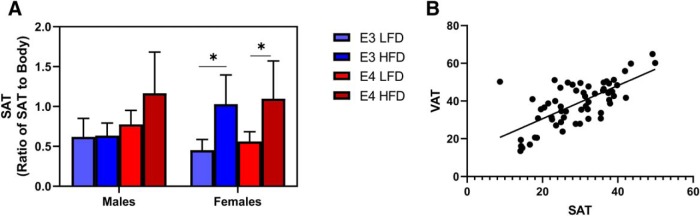
HFD increases SAT in *APOE3* and *APOE4* mice. Within sex and across sex quantification of VAT in *APOE3* and *APOE4* mice (***A***). Correlation of VAT and SAT accumulation (***B***). ***A***, *N* = 5–9, *p < 0.04, three-way ANOVA Tukey’s multiple comparison test. ***B***, *N* = 59, linear regression *R* ^2^ = 0.47, *p* ≤ 0.0001.

The effects of HFD on SAT mirrored its effects on VAT. In females, HFD *APOE3* and *APOE4* mice had similar levels of SAT, and HFD caused an increase in SAT compared to LFD. (*p* < 0.03). In males, SAT accumulation did not differ across genotype or diet (Fig. 4*A*). VAT and SAT levels strongly correlated (*R* ^2^ = 0.47, *p* < 0.0001; Fig. 4*B*).

### Sex affects BAT intensity in *APOE3* and *APOE4* mice

**Figure 5.**
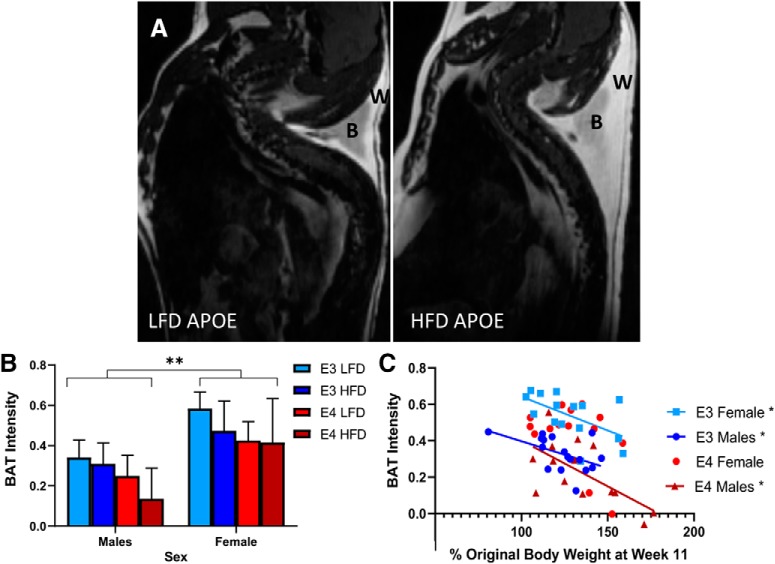
Male *APOE* mice have decreased BAT. Representative image of BAT in the neck in a LFD mouse and HFD mouse (***A***). Comparison of BAT intensity in male and female *APOE3* and *APOE4* mice (***B***). Correlation of genotype and weight to BAT intensity then sex and weight to BAT intensity lines indicate significant correlations (***C***). B, BAT; W, WAT; S, spine, TAT, total adipose tissue; ***B***, *N* = 5–9, **p < 0.004, three-way ANOVA Tukey’s multiple comparison test. ***C***, Linear regression. *APOE3* male (*N* = 17): *R* ^2^ = 0.25, *p* = 0.04; *APOE3* female (*N* = 15): *R* ^2^ = 0.32, *p* = 0.03; *APOE4* male (*N* = 14): *R* ^2^ = 0.42, *p* = 0.01.

BAT is a metabolically active adipose tissue (Schulz and Tseng, 2013). To examine diet associated BAT alterations, we used small rodent MRI and imaged neck BAT (Fig. 5*A*). We examined intensity of BAT, with decreasing intensities indicating the transition to WAT. There was no effect of diet on BAT; however, there were sex differences. Male mice had significantly lower BAT intensities than female mice (∼30%, *p* < 0.004; Fig. 5*B*). The lower BAT intensities indicate less thermogenic energy expenditure which has been implicated in decreased resistance to diet induced obesity (Schulz and Tseng, 2013). We ran correlational analyses to see whether BAT intensity individually correlated with weight gain. In male *APOE4* mice, there was a negative correlation between weight gain and BAT intensity (*R* ^2^ = 0.42, *p* = 0.01; Fig. 5*C*). This was also seen in *APOE3* mice (*R* ^2^ = 0.3, *p* = 0.03, Fig. 5*C*). These correlations indicate weight gain can directly decrease BAT levels, particularly in *APOE3* and male *APOE4* mice.

### Effects of HFD on behavior in *APOE3* and *APOE4* mice

**Figure 6.**
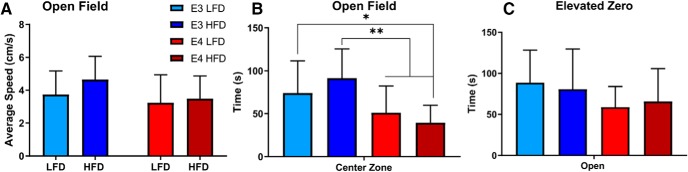
Effects of diet on locomotor activity and anxiety-like behavior. Mice were placed on an open field apparatus and locomotion was recorded. Average speed on OFT analyzed by *APOE* genotype and diet (***A***). Mice were placed on the open field apparatus and EZM and anxiety-like behavior were analyzed. Time spent in the center zone of the OFT (***B***). Time spent in the open arms of the EZM (***C***). ***A–C***, *N* = 13–15 mice. Two-way ANOVA Sidak’s multiple comparison test. ***B***, *p < 0.05, **p < 0.002.

We tested the effects of *APOE* genotype, sex, and diet on cognitive domains in these mice. Since HFD resulted in significant weight gain, we first examined whether movement had been impaired. In the OFT, there were no differences in average speed regardless of diet, sex, or genotype (Fig. 6*A*).

To examine whether HFD induced cognitive alterations in this experiment, we used the OFT, EZM, and BM. OFT and EZM were both used to measure anxiety like behavior. For the OFT, time spent in the center zone was used as a measure of decreased anxiety. We found that all *APOE4* mice spend less time in the center zone than HFD *APOE3* mice (*p < 0.05, **p < 0.002; Fig. 6*B*). There were no differences between sexes (data not shown). EZM, a second measure of anxiety like behavior, did not show any differences by diet, *APOE* genotype, or sex (Fig. 6*C*).

**Figure 7.**
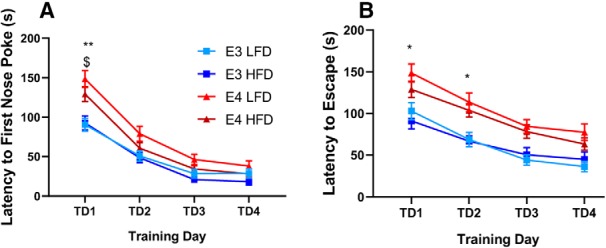
Effects of diet on BM performance. Mice were trained on the BM for four consecutive days, and memory acquisition was measured. Latency to first nose poke of the escape hole (***A***). Latency to escape from the BM (***B***). ***A***, ***B***, *N* = 13–15; *p < 0.03; $*p* < 0.05, **p < 0.004, two-way ANOVA, Sidak’s multiple comparison test.

We used the BM to test spatial learning and memory. The mice were exposed to the maze for four training days and latency to first nose poke and latency to escape were measured each day. For latency to first nose poke, *APOE4* mice showed less learning on training day 1, but matched *APOE3* mice by training day 2 (*p* < 0.03; Fig. 7*A*). For latency to escape, *APOE4* mice were delayed for the first two training days, but by training day 3 the latency to escape matched *APOE3* mice (*p* < 0.03; Fig. 7*B*). There was no effect of diet on either *APOE3* or *APOE4* groups.

## Discussion

Although not as severe as the risk of AD in homozygous *APOE4* carriers, metabolic disturbances caused by a HFD can have a 2- to 3-fold increased risk of cognitive impairment and AD (Gunstad et al., 2007; Whitmer et al., 2008; Profenno et al., 2010). Using an *APOE* knock-in mouse model, we found that *APOE4* increases metabolic disturbances in response to HFD. Furthermore, sex plays a pivotal role in the effects of HFD. We examined differences in weight, baseline glucose levels, glucose intolerance, and adipose tissue disposition and found these to be the most significantly increased in male *APOE4* mice. Female *APOE3* and *APOE4* mice responded similarly to HFD with increased weight, glucose intolerance, and adipose tissue levels. In terms of the types of adipose tissue that increased under the HFD, in males, VAT increases were seen in the *APOE4* mice, but not *APOE3* mice. SAT increases were not seen in *APOE3* or *APOE4* mice. These findings demonstrate that the male *APOE4* group has the greatest accumulation of VAT in response to HFD. In females, VAT and SAT increases were seen in both *APOE3* and *APOE4* mice in response to HFD, indicating there is a similar accumulation in both types of adipose tissue. Throughout the study, female mice had similar metabolic responses to HFD regardless of *APOE* genotype and male *APOE4* mice had more robust metabolic disturbances.

While we cannot directly compare our study to previous studies due differences in the age of mice, diet composition, and length of time on diets, there are similarities across models. With wild-type mice on HFD, male and female mice accumulate similar levels of VAT, but male mice display higher fasting blood glucose levels, insulin levels, and insulin resistance (Macotela et al., 2009; Hwang et al., 2010; Medrikova et al., 2012; Barron et al., 2013). Human studies also showed this pattern: males have increased chances of metabolic syndrome associated with obesity (Hadaegh et al., 2013; Pradhan, 2014). These studies demonstrate that the rodent models can reflect well some effects of HFD on human metabolic disturbances.

Several studies have been conducted in APOE mice on the effects of HFDs, because clinical studies showed *APOE4*-positive individuals have increased risk of metabolic syndrome (Arbones-Mainar et al., 2008; Torres-Perez et al., 2016) and obese *APOE4* carriers have increased metabolic disturbances when compared to *APOE3* carriers (Elosua et al., 2003). Diverse studies, including ours, showed that there were no differences in baseline glucose levels between *APOE3* and *APOE4* mice on HFD, and that HFD induced worse glucose tolerance in *APOE4* mice than in *APOE3* mice (Table 1). These findings support the observed susceptibility of human *APOE4* carriers to metabolic disturbances, underscoring the importance of diet for *APOE4* individuals in particular.

**Table 1.** Studies of the effects of a HFD on *APOE3* and *APOE4* mice

Reference	Onset/duration	Sex	Dietary composition	Metabolic findings	Cognitive findings
Arbones-Mainar et al. (2008)	2 months/8 weeks	Male	21% (w/w) fat and 0.2% (w/w) cholesterol	• Weight gain: E3 HFD > E4 HFD • SAT accumulation: E3 HFD = E4 HFD • VAT accumulation: E3 HFD > E4 HFD • Baseline glucose: E3 HFD = E4 HFD • Glucose intolerance: E4 HFD > E3 HFD	N/A
To et al. (2011)	3 months/32 weeks	Male	60% kcal fat from lard	• Baseline glucose: E3 HFD = E4 HFD • Glucose intolerance: E3 HFD = E4 HFD	N/A
Huebbe et al. (2015)	6–8 weeks/8 months	Female	21% fat from milk	• Weight gain: E3 HFD > E4 HFD	N/A
Arbones-Mainar et al. (2016)	2 months/1–10 months	Male	21% (w/w) fat from milk and 0.2% (w/w) cholesterol	• Weight gain: E3 HFD > E4 HFD • Baseline glucose: E3 HFD = E4 HFD	N/A
Johnson et al. (2017)	9 months/6 months	Female	60% kcal fat from lard	• Weight gain: E3 HFD > E4 HFD • VAT accumulation: E3 HFD > E4 HFD • Baseline glucose: E3 HFD = E4 HFD • Glucose intolerance: E4 HFD > E3 HFD	• Object recognition impairment: E3 HFD = E4 HFD • Cued fear memory: E3 HFD = E4 HFD • Spatial memory: E4 HFD > E3 HFD
Johnson et al. (2019)	9 months/6 months	Female	60% kcal fat from lard	• Weight gain: E3 HFD > E4 HFD • SAT accumulation: E3 HFD = E4 HFD • VAT accumulation: E3 HFD > E4 HFD • Baseline glucose: E3 HFD = E4 HFD • Glucose intolerance: E3 HFD = E4 HFD	• Morris water maze: E3 HFD = E4 HFD

There are several findings that differ from our work (Table 1). Published studies show that *APOE3* mice on several types of HFDs gain more weight when compared to *APOE4* mice (Arbones-Mainar et al., 2008; Huebbe et al., 2015; Johnson et al., 2017), and *APOE3* mice have greater VAT accumulation (Arbones-Mainar et al., 2008; Johnson et al., 2017). In all studies the VAT accumulation reflects the weight gain, with the heavier groups having larger VAT compositions. These differences in VAT accumulation and weight gain across studies could be due to different diet compositions. Our study uses a lard based 45% kcal fat diet; other studies use either a diet where the fat is composed of milk (Arbones-Mainar et al., 2008, 2016; Huebbe et al., 2015) or 60% kcal fat from lard (To et al., 2011; Johnson et al., 2017, 2019). These findings raise the interesting possibility that both the components and the percentage of fat can differentially affect weight gain in *APOE4* carriers. In humans, healthy *APOE4* carriers have lower BMI (Tejedor et al., 2014), although they remain more susceptible to metabolic and cognitive disturbances.

Studies on the effects of HFDs on cognition in non-*APOE* mice showed spatial memory deficits and deficits in other cognitive task including novel object recognition and fear condoning in wild type mouse models (Hwang et al., 2010; Kesby et al., 2015) and AD mouse models (Barron et al., 2013; Knight et al., 2014; Kesby et al., 2015; Lin et al., 2016; Johnson et al., 2017). Studies on the effects of diet on cognition in *APOE* mice showed either equal levels of impairment in *APOE3* and *APOE4* mice on HFD or increased impairment in *APOE4* mice on HFD depending on the behavioral assay (Johnson et al., 2017, 2019). We did not observe robust behavioral effects with our behavioral assays. *APOE4* mice exhibited more anxiety like behavior on the OFT but not on the EZM. With the BM, *APOE4* mice had impairment in spatial learning overall, but diet had only an effect on TD1. Potential effects of diet here may have been obscured by sex differences, which could be addressed in larger cohorts. Previous studies have shown *APOE4* mice have cognitive deficits or decreased neuronal complexity from as early as three months and these deficits remain at later ages such as 21 months (Bour et al., 2008; Rodriguez et al., 2013; Speidell et al., 2019), consistent with APOE genotype dependent deficits seen in our study. Performance in cognitive tasks have differed between sexes also with females performing worse than males (Bour et al., 2008), further emphasizing the need for these behavioral assays to be replicated with a greater number of animals across sexes.

In humans, obesity has been linked to increased risk of AD, cognitive disturbances, and decreases in structural integrity (Enzinger et al., 2005; Gunstad et al., 2007). Middle aged obesity is particularly impactful, associated with increased risk of cognitive disturbances and dementia (Whitmer et al., 2005; Gustafson et al., 2007; Tolppanen et al., 2014). However, higher BMI at later ages is protective (Tolppanen et al., 2014), highlighting a complex relationship between BMI and cognition. Interestingly, while obesity in males is associated with increased susceptibility to metabolic disturbances, obesity in females is associated with increased susceptibility to cognitive changes (Elias et al. 2005; Moser and Pike 2016). Obese females compared to obese males have increased risk of MCI leading to AD, decreased cognitive performances, decreased structural brain integrity (Moser and Pike, 2016). *APOE4* females (compared to *APOE4* males) have an equivalent risk of AD, with a significantly earlier age of onset between 65 and 75 years old (Neu et al., 2017). In mouse studies of *APOE* mice crossed with *5xFAD* (*EFAD*), male obese *E4FAD* mice have higher levels of beta amyloid deposits, glial reactivity, and inflammatory markers compared to non-obese *E4FAD* mice or obese *E3FAD* mice (Moser and Pike, 2017). In female non-obese *EFAD* mice, *APOE4* was associated with higher levels of AD pathology; however the *E3FAD* mice were more affected by HFD, suggesting that the female *E4FAD* mice reached deficits that could not be further exacerbated by diet (Christensen and Pike, 2019). Therefore, while our study does not highlight diet specific behavioral deficits, data greatly support the connection between diet and CNS dysfunction.

Chronic systemic inflammation associated with VAT and the alterations in glucose and insulin may be connected to cognitive disturbances (Jones and Rebeck, 2018). HFD increases systemic inflammation from the increase in VAT (Hotamisligil et al., 1995). This increase in inflammation can both induce metabolic disturbances (Xu et al., 2003) and increase CNS damage (Kempuraj et al., 2017; Varatharaj and Galea, 2017). There is also the possibility that the metabolic disturbances such as glucose intolerance and insulin resistance could more directly lead to CNS damage. Metabolic disturbances have been associated with increased CNS insulin resistance, glucose intolerance (Arnold et al., 2014; Kothari et al., 2017), which can affect brain glucose uptake and neuronal functioning. However, we do not know whether it is the inflammation or metabolic disturbances leading the CNS deficits.

We found that HFD leads to metabolic disturbances particularly in male *APOE4* mice, and in female mice of either *APOE3* or *APOE4* genotypes; however, the underlying mechanisms of this response remain to be defined. Overall, the study implicates *APOE4* positive individuals as more affected by HFD. These connections could affect a large proportion of the population as the increasing rates of obesity increase the risk of metabolic syndrome.

## Synthesis

Reviewing Editor: Jörg Schulz, RWTH Aachen University Decisions are customarily a result of the Reviewing Editor and the peer reviewers coming together and discussing their recommendations until a consensus is reached. When revisions are invited, a fact-based synthesis statement explaining their decision and outlining what is needed to prepare a revision will be listed below. The following reviewer(s) agreed to reveal their identity: Jacob Raber, Lance Johnson.

In the current study, the authors investigate the interaction between two critical risk factors for Alzheimer's Disease (AD), namely APOE4 genotype and diet-induced obesity. The authors employ the commonly used human APOE “targeted replacement” mice, feeding them either a 45% kcal high fat diet (HFD) or a control 10% low fat diet (LFD) for 12 weeks, beginning at 6 months of age. Several metabolic and cognitive/behavioral output measures are reported.

The topic of this study is considered generally interesting and the reviewers acknowledge the careful examination of the interactions between Alzheimer (AD) risk factors ApoE and obesity as reflected by parameters genotype, diet and sex. Nevertheless, the reviewers were concerned about a lack of novelty that was caused by the fact that most of the metabolic and behavioral measures reported in the current study have been reported before, by various groups and in various instances.

There are a number of substantial shortcomings and inconsistencies related to e.g. data interpretation, for example applying different statistics to the same data as in Fig. 2A and Fig. 2E, or lack of satisfactory discussion of risk fators apparently affecting AD differentially, such as obesity in mid-life specifically increasing risk of all cause dementia, while higher BMI in old age actually being protective with regard to AD. This complexity of the relationship between body mass and AD risk is in our view not sufficiently well reflected in the manuscript. It also remains somewhat unclear how the age of mice used in this study relates to this issue.

Likewise, the sample size with n=5 (per genotype, sex, and diet?) appears too small for behavioral or metabolic studies with quite some variance among data; it is not good practice to increase that number retrospectively instead of having calculated the required number “n” a priori in a sample size calculation that would warrant sufficient statistical power.

The reviewers had a number of specific comments that are listed in the following for more detail:

1. The current study involves a different age of mice at the onset of the diets and a different length of diets as compared to previous studies that are been compared and referred. As the experimental conditions are so different, this comparison is invalid. It is a major problem that this is not explicitly stated in this manuscript. This is especially important as discrepant results were found.

2. The number of mice in some groups is only 5. That is not sufficient for behavioral or metabolic studies. The error bars in some of the behavioral figures besides the Barnes maze illustrate this point clearly. It is not totally clear but the assumption is that the n = 5 means per genotype, per sex, and per diet.

3. It is not clearly indicated when the MRI happened compared to the behavioral testing. This is particularly important as the anesthesia used for the MRI might affect the behavioral performance in genotype-, diet-, and sex-dependent ways.

4. The current study involves a different age of mice at the onset of the diets and a different length of diets as compared to previous studies that are been compared and referred. As the experimental conditions are so different, this comparison is invalid. It is a major problem that this is not explicitly stated in this manuscript. This is especially important as discrepant results were found.

5. The number of mice in some groups is only 5. That is not sufficient for behavioral or metabolic studies. The error bars in some of the behavioral figures besides the Barnes maze illustrate this point clearly. It is not totally clear but the assumption is that the n = 5 means per genotype, per sex, and per diet.

6. How is BAT distinguished from WAT in the MRI method described?

7. What are the dimensions of the outer zone in the open field test?

8. Were all behavioral/cognitive tests completed during the 12th week of diet (i.e. mice were still consuming HFD or LFD during testing)?

9. In Figure 1, data is expressed as % original body weight. Can the authors specify whether this is a % of each individual mouse's original weight, or % increase compared to group average, or some other calculation? Is there a reason the authors show the data this way rather than in (g)? Do the E4 mice start at a lower original body weight?

The Reviewer may be mistaken, but in the bar graph in 1C, the E3 difference in males between HFD and LFD looks to the naked eye to be much smaller than the difference in the line graph in 1B.

10. It is not clear what the benefit of showing the same data with different statistics is in Figure 2 A and E. In the Reviewer's opinion, this only serves to confuse the reader.

11. The GTT results in Figure 2 are analyzed in an unusual manner. Typically, repeated measures are used to test for group differences in glucose tolerance. Here, the authors are comparing each groups various time point values to the 0 time value and testing for significance (a measure that is much better reflected by AUC). This cause confusion for the reader, who will likely confuse markers of statistical significance for group differences.

12. Why do the correlations in Figure 2 and 3 use “original body weight” and “30 min” glucose GTT values? Wouldn't week 11 body weight and AUC be more appropriate measures?

13. What is meant by the sentence “Area indicates the ratio of any existing BAT to WAT”?

14. Although there is not a complete consensus, it appears that the majority of previous APOE + HFD studies show larger weight gain and increased adipose tissue mass in E3, not E4 mice, as reported here. The authors do a fair job of comparing and contrasting to previous studies, but a few additional studies that examined the metabolic, cognitive and/or cerebrovascular effects of HFD on APOE are not discussed. The authors should consider commenting on how their own study relates to these additional previous reports. The Reviewer is likely leaving some out (the authors should do a careful check), but examples include: (Huebbe P, Dose J, et al, 2015, Mol Nutr Food Res) (Johnson LA, Torres ER, 2019, J Cereb Blood Flow Metab) (Arbones-Mainar JM, Johnson LA, 2016, Int J Obes) (To AW, Ribe EM, 2011, PLoS One)

15. There appears to be a direct association between APOE genotype and body fatness (Tejedor MT, Garcia-Sobreviela MP, 2014, et al PLoS One). Does this influence interpretation of the current study (and/or previous studies)?

16. By the time the mice have completed the HFD regimen, they should be approximately 9 months old, correct (6 months of age + 12 weeks of diet)? What is known about diet-independent cognitive changes (if any exist) between E3 and E4 mice at this age?

17. The first sentence of the Discussion may not completely reflect the reality of AD odds ratios for APOE4 vs metabolic disturbances. In other words, is the 2-3 fold risk of AD associated with diabetes really comparable to the up to 15-fold risk of AD in E4/E4 individuals?

18. In the paragraph focused on cognition in the discussion, the authors refer to “Studies of the effects of diet on cognition in non-APOE mice”, but at least one cited reference (Johnson, Torres 2017) is a study of human APOE mice. Perhaps the authors were referring to Johnson, Zuloaga et al., 2015, eBiomedicine, or another WT mouse + HFD study, instead? The authors should also probably make a distinction here between HFD studies done in WT mice versus those done in AD mouse models. Along these lines, there are also some studies in EFAD mice (human APOE x 5xFAD) fed a high fat diet (Christenssen and Pike, 2019, FASEB; Moser and Pike, 2017, eNeuro). These experiments seem very relevant to the current study and should probably be discussed.
